# Neonatal resuscitation: A cross-sectional study measuring the readiness of healthcare personnel

**DOI:** 10.12688/f1000research.109110.1

**Published:** 2022-05-13

**Authors:** Martono Tri Utomo, Mahendra Tri Arif Sampurna, Rufina Adelia Widyatama, Visuddho Visuddho, Ivan Angelo Albright, Risa Etika, Dina Angelika, Kartika Darma Handayani, Abyan Irzaldy

**Affiliations:** 1 Department of Pediatrics, Faculty of Medicine, Universitas Airlangga, Dr. Soetomo General Hospital, Surabaya, East Java, 60132, Indonesia; 2 Department of Pediatrics, Faculty of Medicine, Universitas Airlangga, Airlangga Teaching Hospital, Surabaya, East Java, 60115, Indonesia; 3 Faculty of Medicine, Universitas Airlangga, Surabaya, East Java, 60132, Indonesia; 4 Department of Public Health, Erasmus MC, University Medical Center Rotterdam, Rotterdam, South Holland, Postbus 2040, 3000 CA, Indonesia

**Keywords:** Healthcare Personnel, Hospital, Neonate, Readiness, Resuscitation

## Abstract

**Background:** The optimal neonatal resuscitation requires healthcare personnel knowledge and experience. This study aims to assess the readiness of hospitals through its healthcare personnel in performing neonatal resuscitation.

**Methods:** This study was an observational study conducted in May 2021 by distributing questionnaires to nurses, midwives, doctors, and residents to determine the level of knowledge and experience of the subject regarding neonatal resuscitation. We conducted the research in four types of hospitals A, B, C, and D, which are defined by the Regulation of the Minister of Health of the Republic of Indonesia by the capability and availability of medical services. The type A hospital is the hospital with the most complete medical services, while type D hospitals have the least medical services. The comparative analysis between participants’ characteristics and the knowledge or experience score was conducted.

**Results:** The total 123 participants are included in the knowledge questionnaire analysis and 70 participants are included in the resuscitation experience analysis. We showed a significant difference (p = 0.013) of healthcare personnel knowledge between the A type hospital (Median 15.00; Interquartile Range [IQR] 15.00–16.00) and the C type hospital (median 14.50; IQR 12.25–15.75). For the experience, the healthcare personnel of type A and type B hospitals have significantly higher experience scores than the type D hospital (p = 0.014; p = 0.007), but we did not find a significant difference between others type of hospital comparison.

**Conclusions:** In this study, we found that the healthcare personnel from type A and type B hospitals are more experienced than the type D hospital in conducting neonatal resuscitation. We suggest more neonatal resuscitation training to improve the readiness of healthcare personnel from type C and type D hospital.

## Background

Neonatal mortality is one of the standards of neonatal care. Data from developing countries showed that about 4 million babies die in the neonatal period. ^1^ As a developing country, Indonesia also contributes, with the mortality rate reaching 12.4 per 1,000 live births in 2019. ^2^ The right strategy for neonatal referral and the readiness of the hospital must be assessed to decrease the neonatal mortality rate in Indonesia. ^3^^,^^4^

The leading causes of neonatal mortality were prematurity, sepsis, and asphyxia. ^5^^–^^7^ These conditions are often related to the requirement of neonatal resuscitation. ^8^^,^^9^ Neonatal resuscitation is a series of procedures performed to prevent the morbidity and mortality associated with a hypoxic-ischemic tissue injury (brain, heart, kidney) and restore spontaneous breathing and adequate cardiac output. ^10^^,^^11^ The appropriate neonatal resuscitation is believed to increase the survival of neonates and reduce the mortality. ^12^

The neonatal resuscitation service and patient prognosis were strong influence factors in the success of this procedure. Essential tools also must be available and ready to use whenever needed. ^11^^,^^13^ The healthcare personnel which play important roles on the neonatal resuscitation must be prepared by several trainings. ^14^ The trainings are expected to increase the healthcare personnel’s capability and confidence in doing neonatal resuscitation. ^15^

To provide optimal services, healthcare personnel must be prepared with both knowledge and experience. ^16^^–^^18^ Therefore, the factors that are associated with the knowledge and experience of the healthcare personnel need to be discovered. This study aims to assess the readiness of hospitals by analyzing the knowledge and experience of healthcare personnel in performing neonatal resuscitation.

## Methods

### Study design and participants

This research has obtained permission from the Ethics Committee of RSUD Dr. Soetomo Surabaya (Letter of Exemption 0335/LOE/301.4.2/II/2021). The data in this study was collected in May 2021 by distributing questionnaires to nurses, midwives, doctors, and residents to determine the level of knowledge and experience of the subject regarding neonatal resuscitation. The researchers met the participants and gave the explanation about the questionnaire in the pediatrics department of each hospital. Subjects in this study have filled out a statement of consent to be involved in this study. To address potential sources of bias, we invited respondents from all types of hospitals (A-D) to participate in our study.

### Data collection

This study was conducted in May 2021. The participants filled out the questionnaire for knowledge and experience measurement. ^19^^,^^20^ The questionnaire was adopted from Jukkala *et al.* ^20^ study with their permission. They developed questionnaires for measuring knowledge and experience in hospital settings. The questionnaires were then translated into Indonesian. The questionnaire was validated by several experts in neonatal resuscitation, which confirmed it was comprehensible. After that, the questionnaire was disseminated to 10 nurses to assess the validity and reliability using the bivariate correlation test and alpha-cronbach reliability test.

The resuscitation knowledge questionnaire contained 25 statements which are true or false questions. The participants chose the answer by marking either “true” or “false” in the column provided. The correct answer mark is 1 point and the wrong answer mark is 0 point. We obtained the total score for each subject for further analysis. From the 148 respondents, we excluded 25 participants because they did not meet our criteria. Five respondents were excluded because they do not work at a type A to D hospital. A further 20 respondents were excluded because they were co-assistant. Leaving 123 respondents included for the knowledge analysis in this study.

The resuscitation experience questionnaire contained 23 statements regarding neonatal resuscitation. The participants were asked to choose an answer using a Likert scale from one to five indicating from rarely to often doing the job in the statement. The data from each subject was then totaled for further analysis. From the 89 respondents who filled out the experience questionnaire, 19 respondents were excluded because they did not meet our criteria. Three respondents did not work at a type A to D hospital and 16 respondents were co-assistants. Leaving 70 respondents for the resuscitation experience analysis.

### Definitions

Type A–D hospitals are defined by the Regulation of the Minister of Health of the Republic of Indonesia No. 340/MENKES/PER/III/2010. ^21^ The hospital type is classified based on the medical service facilities and their capabilities. For the type A hospitals there must be at least 4 Basic Specialists, 5 Medical Support Specialists, 12 Other Specialists and 13 Sub Specialist Services. Type B hospitals must have at least 4 Basic Specialists, 4 Medical Support Specialists, 8 Other Specialists and 2 Subspecialist Services. Type C hospitals must have at least 4 Basic Specialists and 4 Medical Supporting Specialist Services. Type D hospitals must have at least 2 Basic Specialist Medical Services.

According to the American Academy of Pediatrics (AAP), ^22^ work units in neonatal care are divided into four levels, namely level 1 to level 4. Level 1 is usually carried out to stabilize the condition of term infants with physiologically stable conditions. Level 2 work units are responsible for stabilizing the premature infants and term infants who are physiologically ill. While at level 3, it is necessary to carry out continuous infant stabilization and observation. ^22^ Although there are four levels, in this study we only divided the room into 3 levels. The level 1 consists of the emergency room, baby room, or neonate room, the level 2 consists of a perinatology room, and the level 3 were Neonatal Intensive Care Unit (NICU) or Pediatric Intensive Care Unit (PICU).

### Statistical analysis

We provide tables for each answered question for the knowledge and experience questionnaire. For analysis, we use the average of the total knowledge and experience for the comparative analysis. The continuous data was presented as median and interquartile range (IQR). The Mann-Whitney U test and Kruskal Wallis test were used to compare differences of total knowledge or experiences score between the groups for each factor. The Kruskal Wallis test was used for the multi-categorical data. The Mann-Whitney U test was used for the two-categorical data and the post-hoc analysis. Statistically significant was considered using two-sided α less than 0.05. Statistical analysis was done using the IBM SPSS software (version 23, RRID:SCR_016479).

## Results

### Study participant characteristics

The characteristics of the participants in the study are shown in Table 1. ^47^ For the knowledge questionnaire, the participants mostly worked at type A hospitals (64.2%) and were mostly aged below 30 years. Only one participant was educated in master’s degree and doctoral degree. The participating professions in this study were midwives (37.4%) and nurses (33.3%) and also dominated by women (91.1%). Most of the employees were contract workers, which consists of midwives, nurses, and general practitioners. For the experience questionnaire, the participants mostly worked at type A hospitals (48.6%). Most of the participant’s professions were nurses (45.7%) and the participants were dominated by females (85.7%). Most of the participants had bachelor’s degrees (60%) and the permanent worker (40%) was the most common type of worker.

**Table 1.** Participant demography and characteristics.

Characteristics		Knowledge measured		Resuscitation experience	
		N	%	N	%
Types of Hospital	A	79	64.2	34	48.6
	B	12	12.0	15	21.4
	C	20	16.3	14	20.0
	D	12	9.8	7	10.0
Sex	Male	11	8.9	10	14.3
	Female	112	91.1	60	85.7
Age	<30	69	56.1	27	38.6
	30-40	42	34.1	34	48.6
	40-50	10	8.1	8	11.4
	>50	2	1.6	1	1.4
Education	Associate Degree	67	54.5	26	37.1
	Bachelor Degree	54	43.9	42	60.0
	Master Degree	1	0.8	2	2.9
	Doctoral Degree	1	0.8	0	0.0
Type of Profession	Resident	27	22	23	32.9
	Midwife	46	37.4	6	8.6
	Nurse	41	33.3	32	45.7
	General Practitioners	9	7.3	9	12.9
Work Experience (Years)	<1	54	43.9	13	18.6
	1-5	26	21.1	24	34.3
	5-10	17	13.8	15	21.4
	10-15	11	8.9	7	10.0
	15-20	6	4.9	4	5.7
	>20	9	7.3	7	10.0
Employment Status	Permanent worker	33	26.8	28	40
	Contract worker	64	52.0	16	22.9
	Students	26	21.1	26	37.1
Unit Level	Level 1	64	52.0	24	34.29
	Level 2	4	3.25	5	7.14
	Level 3	55	44.72	41	58.57

### Knowledge questionnaire

Table 2 showed the answers for the knowledge questionnaire. The highest number participants chose false on the statement about chest compression initiation and positive pressure ventilation (87%). Statements about the number of heart rates in infants, infant diagnosis of primary or secondary apnea, the timing of oxygen administration, and the purpose of determining the Apgar score are also considered as hard questions with a high number of participants.

**Table 2.** Answers of knowledge questionnaire. ET: Endotracheal; HR: Heart Rate; PPV: Positive Pressure Ventilation.

No.	Questions	Answers	
		Correct N (%)	False N (%)
1	The size of the ET Tube that is suitable for babies weighing 2,800 grams is 2.5 mm	90 (73.2)	33 (26.8)
2	During chest compressions, the sternum should be pushed in 1.2 to 1.9 cm	72 (58.5)	51 (41.5)
3	Epinephrine administration should be started immediately if HR <60 or 0, with or without previous PPV	30 (24.4)	93 (75.6)
4	Chest compressions and ventilation are performed at least 60 seconds before the second HR evaluation is performed	96 (78.1)	27 (21.2)
5	An ET tube or a 6-F or 8-F suction catheter can be used to suck meconium from the trachea	87 (70.7)	36 (29.3)
6	Delayed drying of a respiratory depressed infant can be used to initiate resuscitation efforts.	98 (79.7)	25 (20.3)
7	PPV in neonates is carried out at a rate of 30-40 times per minute	60 (48.8)	63 (51.2)
8	An orogastric catheter should be inserted if the infant requires balloon and mask ventilation for more than a few minutes.	71 (58.8)	52 (42.3)
9	Chest compressions should be initiated only if the HR is below 60 beats per minute and positive pressure ventilation has been performed for 15-30 seconds	16 (13)	108 (87)
10	In infants showing respiratory effort, the heart rate should be at least 100 beats per minute	11 (9)	112 (91)
11	Poor response to resuscitation is a sign of hypovolemia in neonates	92 (74.8)	31 (25.2)
12	When oxygenating neonates with a mask or oxygen tube, the flowmeter should be set at a dose of 5 lpm	54 (43.9)	69 (56.1)
13	The volume of the mask balloon for neonates should not exceed 750ml	111 (90.2)	12 (9.8)
14	When sucking secretions during intubation, the suction pressure should not exceed -100mmHg	116 (94.3)	7 (5.7)
15	The neonate's nose should be suctioned before the mouth	58 (47.2)	65 (52.8)
16	Each attempt at intubation should be limited to no more than 30 seconds to minimize hypoxia	115 (93.5)	8 (6.5)
17	In neonates, respiratory depression due to narcotics is mostly caused by giving narcotics to the baby's mother within 4 hours before delivery	109 (88.6)	14 (11.4)
18	Expansion of the chest and the presence of breath sounds in both lung fields can be used as indicators of adequate ventilation	120 (97.6)	3 (2.4)
19	When a baby is not breathing at birth, it is very easy to determine whether the baby is primary or secondary apnea	40 (32.5)	83 (67.5)
20	Chest compressions are always accompanied by coordinated positive-pressure ventilation	34 (27.6)	89 (72.4)
21	When secondary apnea occurs, oxygen and stimulation will usually trigger breathing	28 (22.8)	95 (77.2)
22	If the baby's heart rate is >100 and the chest expands, but the baby still shows symptoms of central cyanosis, the most appropriate course of action is to initiate positive pressure ventilation with a mask or an ETT.	82 (66.7)	41 (33.3)
23	Placement of the ET tube can be confirmed by listening for breath sounds in both lung fields.	120 (97.6)	3 (2.4)
24	The APGAR score is used to determine when to start resuscitation and the goals of resuscitation	35 (28.5)	88 (71.5)
25	Complete resuscitation equipment should be available in the delivery room only when there is an indication of the need for resuscitation	114 (92.7)	9 (7.3)

We found a significant difference (p = 0.007) between male (median 17.00; IQR 15.00–18.00) and female (median 15.00; IQR 14.00–16.00) participants as shown in Table 3. The education and type of professional role are important factors on participants knowledge. The students (which is the same population as residents) (median 17.00; IQR 15.00–18.00) have higher knowledge than the permanent (median 15.00; IQR 13.00–16.50) and contract (median 15.00; IQR 15.00–15.00) workers (p = 0.001). The post-hoc analysis showed a significant difference (p = 0.013) of knowledge between the A type hospital (median 15.00; IQR 15.00–16.00) and the C type hospital (median 14.50; IQR 12.25–15.75).

**Table 3.** Comparison between participants characteristic and knowledge score.

Characteristics		Total knowledge score		p-value
		Median	IQR	
Type of Hospital	A	15.00	15.00-16.00	0.119
	B	15.00	13.00-17.00	
	C	14.50	12.25-15.75	
	D	15.00	13.25-16.75	
Sex	Male	17.00	15.00-18.00	0.007 *
	Female	15.00	14.00-16.00	
Age (Year)	<30	15.00	15.00-15.00	0.169
	30-40	15.00	13.75-17.00	
	40-50	16.00	14.75-17.25	
	>50	13.00	12.00-14.00	
Education	Associate Degree	15.00	14.00-15.00	0.009 *
	Bachelor Degree	16.00	14.00-18.00	
	Master Degree	15.00	15.00-15.00	
	Doctoral Degree	18.00	18.00-18.00	
Type of Profession	Resident	17.00	15.00-18.00	0.000 *
	Midwife	15.00	15.00-15.00	
	Nurse	14.00	12.50-16.00	
	General Practitioners	15.00	14.50-17.00	
Work Experience (Year)	<1	15.00	15.00-15.00	0.481
	1-5	16.00	13.75-18.00	
	5-10	15.00	13.00-16.50	
	10-15	14.00	13.00-18.00	
	15-20	14.50	12.75-16.00	
	>20	15.00	14.00-17.50	
Employment Status	Permanent worker	15.00	13.00-16.50	0.001 *
	Contract worker	15.00	15.00-15.00	
	Students	17.00	15.00-18.00	
Unit Level	Level 1	15.00	15.00-15.00	0.410
	Level 2	13.50	10.50-16.50	
	Level 3	15.00	13.00-18.00	
Post Hoc Analysis				
Type of Hospital	A vs B		0.757	
	A vs C		0.013 *	
	A vs D		0.463	
	B vs C		0.261	
	B vs D		0.799	
	C vs D		0.376	

* p-value < 0.05.

### Experience questionnaire

The responses to the knowledge questionnaire were shown in Table 2. The majority of participants rarely performed pulse examinations on umbilical cord (40%). The study also revealed that several participants rarely perform endotracheal suctioning (35.7%), umbilical catheterization (34.3%), take blood through an umbilical vein catheter (47.1%), and administer drugs/fluids through an umbilical catheter (35.7%). Most of them were also not experienced in interpreting the results of neonates' blood gases (27/70; 38.6%) as shown in Table 4.

**Table 4.** Answers of experience questionnaire. PPV: Positive Pressure Ventilation.

No	Questions	Answers N (%)				
		1	2	3	4	5
1.	Provide care to neonates after delivery	11 (15.7)	6 (8.6)	9 (12.9)	9 (12.9)	35 (50)
2.	Drying, positioning, and suctioning the neonate	9 (12.9)	5 (7.1)	8 (11.4)	15 (21.4)	33 (47.1)
3.	Performing suction on the neonate with a suction catheter	9 (12.9)	6 (8.6)	9 (12.9)	16 (22.9)	30 (42.9)
4.	Listening to the newborn's heart rate with a stethoscope	5 (7.1)	6 (8.6)	8 (11.4)	22 (31.4)	29 (41.4)
5.	Feel the pulse through the umbilical cord	28 (40)	11 (15.7)	19 (27.1)	8 (11.4)	4 (5.7)
6.	Turn on the infant warmer before labor begins	7 (10)	3 (4.3)	4 (5.7)	8 (11.4)	48 (68.6)
7.	Assessing the APGAR Score in fit newborns	4 (5.7)	4 (5.7)	9 (12.9)	11 (15.7)	42 (60)
8.	Assessing the APGAR Score in sick newborns	9 (12.9)	6 (8.6)	10 (14.3)	15 (21.4)	30 (42.9)
9.	Inserting an orogastric tube in the neonate	14 (20)	3 (4.3)	10 (14.3)	9 (12.9)	34 (48.6)
10.	Performing airway suctioning in neonates with a suction machine	10 (14.3)	3 (4.3)	6 (8.6)	18 (25.7)	33 (47.1)
11.	Performing endotracheal suctioning in infants with meconium membranes	25 (35.7)	8 (11.4)	13 (18.6)	12 (17.1)	12 (17.1)
12.	Performing PPV with balloons and masks	10 (14.3)	2 (2.9)	16 (22.9)	21 (30)	21 (30)
13.	Perform or assist endotracheal intubation	19 (27.1)	14 (20)	12 (17.1)	9 (12.9)	16 (22.9)
14.	Performing chest compression on the neonate	12 (17.1)	6 (8.6)	19 (27.1)	15 (21.4)	18 (25.7)
15.	Perform/assist umbilical catheter installation	24 (34.3)	9 (12.9)	17 (24.3)	7 (10)	13 (18.6)
16.	Taking blood through an umbilical vein catheter	33 (47.1)	4 (5.7)	16 (22.9)	6 (8.6)	11 (15.7)
17.	Administer medications/fluids through an umbilical catheter	25 (35.7)	9 (12.9)	9 (12.9)	9 (12.9)	18 (25.7)
18.	Interpreting the neonate's blood sugar level	9 (12.9)	7 (10)	11 (15.7)	16 (22.9)	27 (38.6)
19.	Interpreting neonatal blood gas results	27 (38.6)	9 (12.9)	12 (17.1)	10 (14.3)	12 (17.1)
20.	Communicating with family during resuscitation	11 (15.7)	6 (8.6)	15 (21.4)	13 (18.6)	25 (35.7)
21.	Communicating with family after resuscitation	6 (8.6)	8 (11.4)	8 (11.4)	13 (18.6)	35 (50)
22.	Provide emotional support to family during resuscitation	9 (12.9)	5 (7.1)	12 (17.1)	18 (25.7)	26 (37.1)
23.	Provide emotional support to family during resuscitation	7 (10)	3 (4.3)	9 (12.9)	21 (30)	30 (42.9)

Table 5 showed the comparison between each group’s risk factors on participant resuscitation experience. Types of hospital are associated with the experience of the medical profession (p = 0.026) with type B as the highest experience option. In the post-hoc analysis, we know that there are non-significant differences between type A hospital and type B hospitals (p = 0.618). The significant differences for the experience of the healthcare personnel are between A and D hospitals (p = 0.014) and between B and D hospitals (0.007).

**Table 5.** Comparison between participants characteristic and experience score.

Characteristics		Total experience score		p-value
		Median	IQR	
Types of Hospital	A	85.00	70.00-101.00	0.026 *
	B	92.00	81.00-98.00	
	C	81.00	68.25-87.00	
	D	42.00	29.00-75.00	
Sex	Male	74.00	53.25-80.75	0.051
	Female	85.00	70.75-96.75	
Age (Year)	<30	75.00	42.00-86.00	0.022 *
	30-40	85.00	72.25-101.00	
	40-50	91.00	81.50-94.50	
	>50	96.00	96.00-96.00	
Education	Associate Degree	85.00	73.75-93.00	0.453
	Bachelor Degree	83.00	55.75-100.75	
	Master Degree	65.00	60.00-70.00	
Type of Profession	Resident	83.00	70.00-111.00	0.002 *
	Midwife	83.00	54.75-87.00	
	Nurse	89.50	78.75-96.00	
	General Practitioners	42.00	30.00-66.00	
Work Experience (Year)	<1	52.00	33.50-74.50	0.006 *
	1-5	81.00	62.50-105.00	
	5-10	89.00	81.00-104.00	
	10-15	85.00	81.00-98.00	
	15-20	94.00	45.75-101.00	
	>20	90.00	81.00-95.00	
Employment Status	Permanent worker	87.50	78.75-95.75	0.230
	Contract worker	78.00	45.75-88.75	
	Students	77.50	52.00-105.75	
Unit Level	Level 1	74.00	42.00-84.50	0.002 *
	Level 2	78.00	64.50-101.50	
	Level 3	92.00	76.00-99.00	
Post Hoc Analysis				
Type of Hospital	A vs B		0.618	
	A vs C		0.291	
	A vs D		0.014 *	
	B vs C		0.073	
	B vs D		0.007 *	
	C vs D		0.061	

* p-value < 0.05.

We also found asignificant difference (p = 0.022) between the ages, seemingly the older age have more experience on neonatal resuscitation. The type of profession also plays an important role in neonatal resuscitation (p = 0.002). The nurses have the highest experience score (median 89.50; IQR 78.75–96.00) and the general practitioners have the lowest experience score (median 42.00; IQR 30.00–66.00). The longer work experience tended to have a higher experience score (p = 0.006) and the second unit level was the unit level with the lowest experience score compared to the first and third level (p = 0.003).

## Discussion

A high level of knowledge and experience of neonatal care is the key to the success of the resuscitation team. ^12^^,^^15^^,^^20^ Our study describes the knowledge and experience of the health care provider in tertiary hospitals in Indonesia. We found the readiness of healthcare personnel was associated with the type of hospital. We found that medical personnel in the type A hospital have better knowledge that the type C hospital. For the experience, the type A and type B hospitals showed more experienced healthcare personnel than the type D hospital. This study also reveals several factors that influence knowledge and experience. Hence, this study may be used as a reference in the neonatal resuscitation guidelines or policies.

Neonatal resuscitation is an action that requires decisive skill which is obtained by knowledge and experience. ^23^ The neonatal resuscitation team training must be conducted in sufficient time to ensure the capability for the healthcare personnel. ^11^^,^^23^ The availability of tools is also an important factor of hospital readiness to perform this procedure. ^13^ Type A or type B hospitals have more qualified facilities to perform the neonatal resuscitation. This is the reason why type A and type B hospitals have better experience in performing neonatal resuscitation than type D hospitals. This also indicates that neonatal resuscitation must be done at the type A or type B hospitals since they are more ready to perform the procedure.

Residents have the highest knowledge score among other types of professions. The students also have the highest knowledge score, since they mostly consist of residents. Knowledge of neonatal resuscitation is a competency that must be mastered by residents during their education as a prospective specialist. ^24^^,^^25^ Residents have the responsibility to plan treatment according to the patient's condition. Even with supervision, residents are actually expected to have extensive knowledge about the causes, diagnosis, prognosis, complication, and management of neonates. ^26^^,^^27^

We found that nurses have the best experience scores among other types of professions. Nursing is a profession that is directly involved in providing services to the patients. ^16^^,^^28^^,^^29^ In the tertiary hospitals, where there are very large numbers of patients, doctors are often more involved in planning patient management. In this study, almost all general practitioners are young doctors, who just registered as the internship doctors. That may be the reason for their lack of experience. However, the right strategy needs to be implemented to improve the experience for general practitioners, since they will help in handling the newborns later. ^30^

Previous studies have reported the relation between the age and the experience of neonatal resuscitation. ^18^ Experience will be gained after several times doing and practicing the procedure. ^31^^,^^32^ This is also the reason why work experience has a significant relation to the experience score. Experienced practitioners were found to be more confident in performing actions on neonatal patients. ^33^^,^^34^

We found a significant difference between unit level and the total experience score. Higher unit levels have higher total experience scores. This is because at the level 1 unit, the baby being treated is a normal baby, while the higher level of care is related to more complications suffered by the babies. ^22^^,^^35^ The more difficult procedure may not be conducted at the unit level 1 and level 2, while this procedure is often held in the unit level 3. ^22^ However, we did not find any difference in knowledge between the three unit levels. Although most of the treatment in the level one unit is a normal baby, knowledge of signs of severity and early treatment is important at all levels. ^36^

Additional training using The Newborn Resuscitation Manual from the United Kingdom with skill demonstrations and scenarios using mannequins have been proven to increase the level of knowledge of nurses, doctors, resident doctors, and specialists in Northern Nigeria. ^19^ To increase personal experience, the health care providers need to practice each step of resuscitation. ^37^ Routine training may be an important indicator in determining the hospital's readiness to conduct the neonatal resuscitation. ^38^ Training on the steps of neonatal resuscitation, especially in the steps of palpating umbilical cord pulse, endotracheal suctioning, endotracheal intubation, umbilical catheter placement, taking blood through an umbilical vein catheter, administering drugs/fluids through an umbilical catheter, and interpreting neonatal blood gas results, must be a concern and require more intense training since most of the research subjects in this study rarely perform them. ^39^^,^^40^

Endotracheal intubation in neonates is rarely done because of the high level of difficulty and high risk of an adverse event for the procedure. ^40^^,^^41^ Even for the skilled healthcare personnel, sometimes they still need to do several attempts until the intubation can enter the trachea of the neonate. ^38^^,^^41^ The placement of an umbilical catheter, blood collection, and administration of drugs through the umbilical vein are rarely done, possibly because of its potential to be a risk factor of sepsis. ^42^^,^^43^ More practice with evaluation are needed to increase the healthcare personnel confidence in doing the neonatal resuscitation. ^44^^–^^46^

### Research strengths and limitation

These findings may provide additional information to the guidelines of healthcare personnel training and qualifications. The participants joined this research voluntarily and were given brief socialization to make sure of the comprehension of the questionnaire to decrease risk of bias. However, several limitations exist in our study. First, the number of research subjects was reduced by the COVID-19 pandemic. We did consecutive sampling rather than random sampling which is more applicable. Second, we did not assess how many times the participants have joined the neonatal resuscitation training. The previous training may be associated with the knowledge and experience score of the participants.

## Conclusion

The success of neonatal resuscitation is influenced by the readiness of the hospital, which can be seen through indicators of the level of knowledge and experience of the healthcare personnel. In this study, we found that the healthcare personnel from type A and type B hospitals are more experienced than the type D hospital in conducting neonatal resuscitation. We suggest that the type D hospital or other primary care must refer the neonate if there is the need for neonatal resuscitation. Additional neonatal resuscitation training is necessary to increase the knowledge and experience of the healthcare personnel. Finally, larger observational studies with multi-center approaches need to be conducted to confirm our findings.

## Data availability

### Underlying data

Figshare: Neonatal Resuscitation: Measuring The Readiness of Healthcare Personnel, https://doi.org//10.6084/m9.figshare.18865418. ^47^

The project contains the following underlying data:

• Experience.sav
• Knowledge.sav

Data are available under the terms of the Creative Commons Attribution 4.0 International license (CC-BY 4.0).
