# Neonatal resuscitation: A cross-sectional study measuring the readiness of healthcare personnel

**DOI:** 10.12688/f1000research.109110.2

**Published:** 2023-05-22

**Authors:** Martono Tri Utomo, Mahendra Tri Arif Sampurna, Rufina Adelia Widyatama, Visuddho Visuddho, Ivan Angelo Albright, Risa Etika, Dina Angelika, Kartika Darma Handayani, Abyan Irzaldy

**Affiliations:** 1 Department of Pediatrics, Faculty of Medicine, Universitas Airlangga, Dr. Soetomo General Hospital, Surabaya, East Java, 60132, Indonesia; 2 Department of Pediatrics, Faculty of Medicine, Universitas Airlangga, Airlangga Teaching Hospital, Surabaya, East Java, 60115, Indonesia; 3 Faculty of Medicine, Universitas Airlangga, Surabaya, East Java, 60132, Indonesia; 4 Department of Public Health, Erasmus MC, University Medical Center Rotterdam, Rotterdam, South Holland, Postbus 2040, 3000 CA, Indonesia

**Keywords:** Healthcare Personnel, Hospital, Neonate, Readiness, Resuscitation

## Abstract

**Background:** Optimal neonatal resuscitation requires knowledge and experience on the part of healthcare personnel. This study aims to assess the readiness of hospital healthcare personnel to perform neonatal resuscitation.

**Methods:** This was an observational study conducted in May 2021 by distributing questionnaires to nurses, midwives, doctors, and residents to determine the level of knowledge and experience of performing neonatal resuscitation. Questionnaires were adapted from prior validated questionnaires by Jukkala AM and Henly SJ. We conducted the research in four types of hospitals A, B, C, and D, which are defined by the Regulation of the Minister of Health of the Republic of Indonesia. Type A hospitals have the most complete medical services, while type D hospitals have the least medical services. The comparative analysis between participants’ characteristics and the knowledge or experience score was conducted.

**Results: **A total of 123 and 70 participants were included in the knowledge and experience questionnaire analysis, respectively. There was a significant difference (p = 0.013) in knowledge of healthcare personnel between the type A hospital (median 15.00; Interquartile Range [IQR] 15.00–16.00) and type C hospital (median 14.50; IQR 12.25–15.75). In terms of experience, the healthcare personnel of type A (median 85.00; IQR 70.00-101.00) and type B (median 92.00; IQR 81.00-98.00) hospitals had significantly (p =0,026) higher experience scores than the type D (median 42.00; IQR 29.00-75.00) hospital, but we did not find a significant difference between other type of hospitals.

**Conclusions:** In this study, we found that the healthcare personnel from type A and type B hospitals are more experienced than those from type D hospitals in performing neonatal resuscitation. We suggest that a type D hospital should refer the neonate to a type A or type B hospital if there is sufficient time in cases of risk at need for resuscitation.

## Background

To decrease the neonatal mortality in developing countries, there is an urgent need to improve the neonatal care. Data from developing countries showed that about 4 million babies die in the neonatal period. ^1^ Among these developing countries, Indonesia had a mortality rate reaching 12.4 per 1,000 live births in 2019. ^2^ The optimal strategy for neonatal referral and readiness of the hospital must be assessed and shored up to decrease the neonatal mortality rate in Indonesia. ^3^^,^^4^

The leading causes of neonatal mortality were prematurity, sepsis, and asphyxia. ^5^^–^^7^ These conditions often necessitate neonatal resuscitation. ^8^^,^^9^ Neonatal resuscitation involves a series of procedures performed to prevent the morbidity and mortality associated with a hypoxic-ischemic tissue injury (brain, heart, kidney) and restore spontaneous breathing and adequate cardiac output. ^10^^,^^11^ Appropriate neonatal resuscitation is believed to increase the survival of neonates and reduce the mortality. ^12^

Neonatal resuscitation service and patient prognosis are strong factors influencing the success of this procedure. Essential equipment must also be readily available for use, whenever needed. ^11^^,^^13^ The healthcare personnel which play important roles on the neonatal resuscitation must be prepared with comprehensive training. ^14^ Training is expected to increase the capability and confidence of healthcare personnel in performing neonatal resuscitation. ^15^

To provide optimal services, healthcare personnel must be prepared with both knowledge and experience. ^16^^–^^18^ Therefore, the factors that are associated with the knowledge and experience of the healthcare personnel need to be assessed and actions taken, to increase the level of these qualities. This study aims to assess the readiness of hospitals by analyzing the knowledge and experience of healthcare personnel in performing neonatal resuscitation.

## Methods

### Study design and participants

The data in this study was collected in May 2021 by distributing questionnaires to nurses, midwives, doctors, and residents to determine the level of knowledge and experience of the subject regarding neonatal resuscitation. The researchers met the participants at the pediatrics department of each hospital and explained the questionnaire. The participants filled out a statement of consent to be participating in this study. To address one potential source of bias, we invited respondents from all types of hospitals (A-D) to participate in our study.

### Data collection

This study was conducted in May 2021. The participants filled out the questionnaire for knowledge and experience measurement. The questionnaire was adapted from the study of Jukkala AM and Henly SJ with permission. ^19^^,^^20^ They developed questionnaires for measuring knowledge and experience in hospital settings. The questionnaires were then translated into Indonesian. The questionnaire was then circulated to 10 nurses to assess its validity and reliability using the bivariate correlation test and Cronbach’s alpha reliability test.

The resuscitation knowledge questionnaire contained 25 statements which are true or false questions. The participants chose the answer by marking either “true” or “false” in the corresponding column. The mark for each correct answer was 1 point, and 0 points for each incorrect answer. We obtained the total score of each participant for further analysis.

The resuscitation experience questionnaire contained 23 statements regarding neonatal resuscitation. The participants were asked to choose an answer using a Likert scale from one to five to indicate how frequently they did each job from ‘rarely’ to ‘often’. The data from each subject was then summed for further analysis.

### Definitions

Type A–D hospitals are defined by the Regulation of the Minister of Health of the Republic of Indonesia No. 340/MENKES/PER/III/2010. ^21^ The hospital type is classified based on the medical service facilities and their capabilities. For the type A hospitals there must be at least 4 Basic Specialists, 5 Medical Support Specialists, 12 Other Specialists and 13 Sub-specialist Services. Type B hospitals must have at least four Basic Specialists, four Medical Support Specialists, eight Other Specialists, and two Sub-specialist Services. Type C hospitals must have at least 4 Basic Specialists and 4 Medical Support Specialist Services. Finally, type D hospitals must have at least 2 Basic Specialist Medical Services.

According to the American Academy of Pediatrics (AAP), ^22^ work units in neonatal care are divided into four levels, namely level 1 to level 4. Level 1 units stabilizes the condition of term infants with physiologically stable conditions. Level 2 work units are responsible for stabilizing the premature infants and term infants who are physiologically ill. Level 3, it is necessary to carry out continuous infant stabilization and observation. Level 4 meet all three level capabilities, plus have experience caring for the most complex and critically ill newborns. ^22^ However, in this study we only have three level of NICU. Level 1 consisted of the emergency room, baby room, or neonate room; Level 2 consisted of a perinatology room; and Level 3 consisted of a Neonatal Intensive Care Unit (NICU).

### Statistical analysis

We provide tables for each answered question for the knowledge and experience questionnaire. For analysis, we use the average of the total knowledge and experience scores for the comparative analysis. The continuous data was presented as median and interquartile range (IQR). The Mann-Whitney U test and Kruskal-Wallis test were used to compare differences of total knowledge or experiences score between the groups for each factor. The Kruskal-Wallis test was used for the multi-categorical data. The Mann-Whitney U test was used for the two-categorical data and the post-hoc analysis. Statistically significant was considered using two-sided α less than 0.05. Statistical analysis was done using the IBM SPSS software (version 23, RRID:SCR_016479).

## Results

### Study participant characteristics

The characteristics of the participants in the study are shown in Table 1. ^47^ Total 123 respondent fill the knowledge questionnaire and 70 respondent fill the experience questionnaire. The respondents of knowledge questionnaire mostly worked at type A hospitals (64.2%) and were mostly aged below 30 years. Only one participant was educated in master’s degree and doctoral degree. The participating professions in this study were midwives (37.4%) and nurses (33.3%) and also dominated by women (91.1%). Most of the employees were contract workers, which consists of midwives, nurses, and general practitioners. For the experience questionnaire, the participants mostly worked at type A hospitals (48.6%). Most of the participant’s professions were nurses (45.7%) and the participants were predominantly females (85.7%). Most of the participants had bachelor’s degrees (60%) and the permanent worker (40%) was the most common type of worker.

**Table 1.** Characteristics and demographics of participants.

Characteristics		Knowledge measured		Resuscitation experience	
		N	%	N	%
Types of Hospital	A	79	64.2	34	48.6
	B	12	12.0	15	21.4
	C	20	16.3	14	20.0
	D	12	9.8	7	10.0
Sex	Male	11	8.9	10	14.3
	Female	112	91.1	60	85.7
Age	<30	69	56.1	27	38.6
	30-40	42	34.1	34	48.6
	40-50	10	8.1	8	11.4
	>50	2	1.6	1	1.4
Education	Associate Degree	67	54.5	26	37.1
	Bachelor Degree	54	43.9	42	60.0
	Master Degree	1	0.8	2	2.9
	Doctoral Degree	1	0.8	0	0.0
Type of Profession	Resident	27	22	23	32.9
	Midwife	46	37.4	6	8.6
	Nurse	41	33.3	32	45.7
	General Practitioners	9	7.3	9	12.9
Work Experience (Years)	<1	54	43.9	13	18.6
	1-5	26	21.1	24	34.3
	5-10	17	13.8	15	21.4
	10-15	11	8.9	7	10.0
	15-20	6	4.9	4	5.7
	>20	9	7.3	7	10.0
Employment Status	Permanent worker	33	26.8	28	40
	Contract worker	64	52.0	16	22.9
	Students	26	21.1	26	37.1
Unit Level	Level 1	64	52.0	24	34.29
	Level 2	4	3.25	5	7.14
	Level 3	55	44.72	41	58.57

In both questionnaires, the profession may confound education, work experience, employment status, and unit level. Most of the residents were bachelor degree, below 5 years experience, were students, and on the neonatal care level 3. Most general practitioners have bachelor degrees, below 5 years experience, were contract workers, and on the neonatal care level 1. Most midwives have associate degrees, below 1 years experience, were contract workers, and on the neonatal care level 1. For nurses, they distribute well in variables.

### Knowledge questionnaire

Table 2 showed the answers to the knowledge questionnaire. The highest number participants chose incorrect on the statement about chest compression initiation and positive pressure ventilation (87%). Statements about the number of heart rates in infants, infant diagnosis of primary or secondary apnea, the timing of oxygen administration, and the purpose of determining the Apgar score were also considered difficult questions by many participants.

**Table 2.** Answers to knowledge questionnaire. The question number 2,3,4,5,6,10,15,19,21,22,24,25 were “reverse” questions which must be answered by false to get correct. ET: Endotracheal; HR: Heart Rate; PPV: Positive Pressure Ventilation.

No.	Questions	Answers	
		Correct N (%)	Incorrect N (%)
1	The size of the ET Tube that is suitable for babies weighing 2,800 grams is 2.5 mm	90 (73.2)	33 (26.8)
2	During chest compressions, the sternum should be pushed in 1.2 to 1.9 cm	72 (58.5)	51 (41.5)
3	Epinephrine administration should be started immediately if HR <60 or 0, with or without previous PPV	30 (24.4)	93 (75.6)
4	Chest compressions and ventilation are performed at least 60 seconds before the second HR evaluation is performed	96 (78.1)	27 (21.2)
5	An ET tube or a 6-F or 8-F suction catheter can be used to suck meconium from the trachea	87 (70.7)	36 (29.3)
6	Delayed drying of a respiratory depressed infant can be used to initiate resuscitation efforts.	98 (79.7)	25 (20.3)
7	PPV in neonates is carried out at a rate of 30-40 times per minute	60 (48.8)	63 (51.2)
8	An orogastric catheter should be inserted if the infant requires balloon and mask ventilation for more than a few minutes.	71 (58.8)	52 (42.3)
9	Chest compressions should be initiated only if the HR is below 60 beats per minute and positive pressure ventilation has been performed for 15-30 seconds	16 (13)	108 (87)
10	In infants showing respiratory effort, the heart rate should be at least 100 beats per minute	11 (9)	112 (91)
11	Poor response to resuscitation is a sign of hypovolemia in neonates	92 (74.8)	31 (25.2)
12	When oxygenating neonates with a mask or oxygen tube, the flowmeter should be set at a dose of 5 lpm	54 (43.9)	69 (56.1)
13	The volume of the mask balloon for neonates should not exceed 750ml	111 (90.2)	12 (9.8)
14	When sucking secretions during intubation, the suction pressure should not exceed -100mmHg	116 (94.3)	7 (5.7)
15	The neonate's nose should be suctioned before the mouth	58 (47.2)	65 (52.8)
16	Each attempt at intubation should be limited to no more than 30 seconds to minimize hypoxia	115 (93.5)	8 (6.5)
17	In neonates, respiratory depression due to narcotics is mostly caused by giving narcotics to the baby's mother within 4 hours before delivery	109 (88.6)	14 (11.4)
18	Expansion of the chest and the presence of breath sounds in both lung fields can be used as indicators of adequate ventilation	120 (97.6)	3 (2.4)
19	When a baby is not breathing at birth, it is very easy to determine whether the baby is primary or secondary apnea	40 (32.5)	83 (67.5)
20	Chest compressions are always accompanied by coordinated positive-pressure ventilation	34 (27.6)	89 (72.4)
21	When secondary apnea occurs, oxygen and stimulation will usually trigger breathing	28 (22.8)	95 (77.2)
22	If the baby's heart rate is >100 and the chest expands, but the baby still shows symptoms of central cyanosis, the most appropriate course of action is to initiate positive pressure ventilation with a mask or an ET tube.	82 (66.7)	41 (33.3)
23	Placement of the ET tube can be confirmed by listening for breath sounds in both lung fields.	120 (97.6)	3 (2.4)
24	The APGAR score is used to determine when to start resuscitation and the goals of resuscitation	35 (28.5)	88 (71.5)
25	Complete resuscitation equipment should be available in the delivery room only when there is an indication of the need for resuscitation	114 (92.7)	9 (7.3)

We found a significant difference (p = 0.007) between male (median 17.00; IQR 15.00–18.00) and female (median 15.00; IQR 14.00–16.00) participants as shown in Table 3. The education and type of professional role were important factors on participants knowledge. The students (which was the same population as residents) (median 17.00; IQR 15.00–18.00) had higher knowledge than the permanent (median 15.00; IQR 13.00–16.50) and contract (median 15.00; IQR 15.00–15.00) workers (p = 0.001). The post-hoc analysis showed a significant difference (p = 0.013) of knowledge between the type A (median 15.00; IQR 15.00–16.00) and the type C hospitals (median 14.50; IQR 12.25–15.75).

**Table 3.** Comparison between participant characteristics and knowledge score.

Characteristics		Total knowledge score		p-value
		Median	IQR	
Type of Hospital	A	15.00	15.00-16.00	0.119
	B	15.00	13.00-17.00	
	C	14.50	12.25-15.75	
	D	15.00	13.25-16.75	
Sex	Male	17.00	15.00-18.00	0.007 *
	Female	15.00	14.00-16.00	
Age (Year)	<30	15.00	15.00-15.00	0.169
	30-40	15.00	13.75-17.00	
	40-50	16.00	14.75-17.25	
	>50	13.00	12.00-14.00	
Education	Associate Degree	15.00	14.00-15.00	0.009 *
	Bachelor Degree	16.00	14.00-18.00	
	Master Degree	15.00	15.00-15.00	
	Doctoral Degree	18.00	18.00-18.00	
Type of Profession	Resident	17.00	15.00-18.00	0.000 *
	Midwife	15.00	15.00-15.00	
	Nurse	14.00	12.50-16.00	
	General Practitioners	15.00	14.50-17.00	
Work Experience (Year)	<1	15.00	15.00-15.00	0.481
	1-5	16.00	13.75-18.00	
	5-10	15.00	13.00-16.50	
	10-15	14.00	13.00-18.00	
	15-20	14.50	12.75-16.00	
	>20	15.00	14.00-17.50	
Employment Status	Permanent worker	15.00	13.00-16.50	0.001 *
	Contract worker	15.00	15.00-15.00	
	Students	17.00	15.00-18.00	
Unit Level	Level 1	15.00	15.00-15.00	0.410
	Level 2	13.50	10.50-16.50	
	Level 3	15.00	13.00-18.00	
Post Hoc Analysis				
Type of Hospital	A vs B		0.757	
	A vs C		0.013 *	
	A vs D		0.463	
	B vs C		0.261	
	B vs D		0.799	
	C vs D		0.376	

* p-value < 0.05.

### Experience questionnaire

The responses to the experience questionnaire were shown in Table 4. Most participants rarely performed pulse examinations of the umbilical cord (40%). The study also revealed that several participants rarely perform endotracheal suctioning (35.7%), umbilical catheterization (34.3%), took blood through an umbilical vein catheter (47.1%), and administer drugs/fluids through an umbilical catheter (35.7%). Most participants were also not experienced in interpreting the results of neonatal’ blood gases (27/70; 38.6%) as shown in Table 4.

**Table 4.** Answers to experience questionnaire. The Experience Questionnaire using a Likert scale from one to five indicate ‘very rare’ to ‘very often’. PPV: Positive Pressure Ventilation.

No	Questions	Answers N (%)				
		1	2	3	4	5
1.	Provide care to neonates after delivery	11 (15.7)	6 (8.6)	9 (12.9)	9 (12.9)	35 (50)
2.	Drying, positioning, and suctioning the neonate	9 (12.9)	5 (7.1)	8 (11.4)	15 (21.4)	33 (47.1)
3.	Performing suction on the neonate with a suction catheter	9 (12.9)	6 (8.6)	9 (12.9)	16 (22.9)	30 (42.9)
4.	Listening to the newborn's heart rate with a stethoscope	5 (7.1)	6 (8.6)	8 (11.4)	22 (31.4)	29 (41.4)
5.	Feel the pulse through the umbilical cord	28 (40)	11 (15.7)	19 (27.1)	8 (11.4)	4 (5.7)
6.	Turn on the infant warmer before labor begins	7 (10)	3 (4.3)	4 (5.7)	8 (11.4)	48 (68.6)
7.	Assessing the APGAR Score in fit newborns	4 (5.7)	4 (5.7)	9 (12.9)	11 (15.7)	42 (60)
8.	Assessing the APGAR Score in sick newborns	9 (12.9)	6 (8.6)	10 (14.3)	15 (21.4)	30 (42.9)
9.	Inserting an orogastric tube in the neonate	14 (20)	3 (4.3)	10 (14.3)	9 (12.9)	34 (48.6)
10.	Performing airway suctioning in neonates with a suction machine	10 (14.3)	3 (4.3)	6 (8.6)	18 (25.7)	33 (47.1)
11.	Performing endotracheal suctioning in infants with meconium membranes	25 (35.7)	8 (11.4)	13 (18.6)	12 (17.1)	12 (17.1)
12.	Performing PPV with balloons and masks	10 (14.3)	2 (2.9)	16 (22.9)	21 (30)	21 (30)
13.	Perform or assist endotracheal intubation	19 (27.1)	14 (20)	12 (17.1)	9 (12.9)	16 (22.9)
14.	Performing chest compression on the neonate	12 (17.1)	6 (8.6)	19 (27.1)	15 (21.4)	18 (25.7)
15.	Perform/assist umbilical catheter installation	24 (34.3)	9 (12.9)	17 (24.3)	7 (10)	13 (18.6)
16.	Taking blood through an umbilical vein catheter	33 (47.1)	4 (5.7)	16 (22.9)	6 (8.6)	11 (15.7)
17.	Administer medications/fluids through an umbilical catheter	25 (35.7)	9 (12.9)	9 (12.9)	9 (12.9)	18 (25.7)
18.	Interpreting the neonate's blood sugar level	9 (12.9)	7 (10)	11 (15.7)	16 (22.9)	27 (38.6)
19.	Interpreting neonatal blood gas results	27 (38.6)	9 (12.9)	12 (17.1)	10 (14.3)	12 (17.1)
20.	Communicating with family during resuscitation	11 (15.7)	6 (8.6)	15 (21.4)	13 (18.6)	25 (35.7)
21.	Communicating with family after resuscitation	6 (8.6)	8 (11.4)	8 (11.4)	13 (18.6)	35 (50)
22.	Provide emotional support to family during resuscitation	9 (12.9)	5 (7.1)	12 (17.1)	18 (25.7)	26 (37.1)
23.	Provide emotional support to family during resuscitation	7 (10)	3 (4.3)	9 (12.9)	21 (30)	30 (42.9)

Table 5 shows the comparison between risk factors of each group in terms off resuscitation experience. The type of hospital was associated with the experience of the medical profession (p = 0.026) with type B having the highest experience. In the post-hoc analysis, we know that there are non-significant differences between type A hospital and type B hospitals (p = 0.618). The significant differences for the experience of the healthcare personnel are between A and D hospitals (p = 0.014) and between B and D hospitals (0.007).

**Table 5.** Comparison between participant characteristics and experience score.

Characteristics		Total experience score		p-value
		Median	IQR	
Types of Hospital	A	85.00	70.00-101.00	0.026 *
	B	92.00	81.00-98.00	
	C	81.00	68.25-87.00	
	D	42.00	29.00-75.00	
Sex	Male	74.00	53.25-80.75	0.051
	Female	85.00	70.75-96.75	
Age (Year)	<30	75.00	42.00-86.00	0.022 *
	30-40	85.00	72.25-101.00	
	40-50	91.00	81.50-94.50	
	>50	96.00	96.00-96.00	
Education	Associate Degree	85.00	73.75-93.00	0.453
	Bachelor Degree	83.00	55.75-100.75	
	Master Degree	65.00	60.00-70.00	
Type of Profession	Resident	83.00	70.00-111.00	0.002 *
	Midwife	83.00	54.75-87.00	
	Nurse	89.50	78.75-96.00	
	General Practitioners	42.00	30.00-66.00	
Work Experience (Year)	<1	52.00	33.50-74.50	0.006 *
	1-5	81.00	62.50-105.00	
	5-10	89.00	81.00-104.00	
	10-15	85.00	81.00-98.00	
	15-20	94.00	45.75-101.00	
	>20	90.00	81.00-95.00	
Employment Status	Permanent worker	87.50	78.75-95.75	0.230
	Contract worker	78.00	45.75-88.75	
	Students	77.50	52.00-105.75	
Unit Level	Level 1	74.00	42.00-84.50	0.002 *
	Level 2	78.00	64.50-101.50	
	Level 3	92.00	76.00-99.00	
Post Hoc Analysis				
Type of Hospital	A vs B		0.618	
	A vs C		0.291	
	A vs D		0.014 *	
	B vs C		0.073	
	B vs D		0.007 *	
	C vs D		0.061	

* p-value < 0.05.

We also found asignificant difference (p = 0.022) between the age groups, seemingly the older participants had more experience on neonatal resuscitation. Profession type also played an important role in neonatal resuscitation (p = 0.002). The nurses had the highest experience score (median 89.50; IQR 78.75–96.00) and the general practitioners have the lowest experience score (median 42.00; IQR 30.00–66.00). Longer work experience tended to be associated with a higher experience score (p = 0.006) and the second unit level was the unit level with the lowest experience score compared to the first and third level (p = 0.003).

## Discussion

A high level of knowledge and experience of neonatal care is the key to the success of any resuscitation team. ^12^^,^^15^^,^^20^ Our study evaluated the knowledge and experience of the health care provider in tertiary hospitals in Indonesia. We found the readiness of healthcare personnel was associated with the type of hospital. We found that medical personnel in the Type A hospital have better knowledge that those in Type C hospital. In terms of experience, the type A and type B hospitals had more experienced healthcare personnel than the type D hospitals. This study also reveals several factors that influence knowledge and experience. Hence, this study may be used as a reference for developing neonatal resuscitation guidelines or policies.

Neonatal resuscitation requires skill of being, which is a product of knowledge and experience. The training of neonatal resuscitation team must be conducted in sufficient depth and refreshed frequently to ensure the ongoing capability and readiness of healthcare personnel. ^11^^,^^23^ The availability of tools is also an important factor of hospital readiness to perform this procedure. ^13^ Type A or type B hospitals had more qualified facilities to perform the neonatal resuscitation. This is the reason why type A and type B hospitals have better experience in performing neonatal resuscitation than type D hospitals. This also indicates that neonatal resuscitation should ideally be performed at type A or type B hospitals since they are more ready to perform the procedure.

Residents have the highest knowledge score among the professions. The students group also have the highest knowledge score, since they mostly consist of residents. Knowledge of neonatal resuscitation is a competency that must be mastered by residents during their education as a prospective specialist. ^24^^,^^25^ Residents have the responsibility to plan treatment according to the patient's condition. Even with supervision, residents are actually expected to have extensive knowledge about the causes, diagnosis, prognosis, complication, and management of neonates. ^26^^,^^27^

We found that nurses have the best experience scores among other types of professions. Nursing is a profession that is directly involved in providing services to the patients. ^16^^,^^28^^,^^29^ In the tertiary hospitals, where there are very large numbers of patients, doctors are often more involved in planning patient management. In this study, almost all general practitioners are young doctors, who just registered as the intern doctors. That may be the reason for their lack of experience. However, the right strategy needs to be implemented to improve the experience for general practitioners, since they will help in handling the newborns. ^30^

Previous studies have reported the relation between the age and the experience in neonatal resuscitation. ^18^ Experience will be gained after several times doing and practicing the procedure. ^31^^,^^32^ This is also the reason why work experience has a significant relation to the experience score. Experienced practitioners were found to be more confident in performing procedures on neonatal. ^33^^,^^34^

We found a significant difference between unit level and the total experience score. Higher unit levels have higher total experience scores. This is because at the level 1 unit, the baby being treated is a normal baby, while the higher level of care is related to more complications suffered by the babies. ^22^^,^^35^ The more difficult procedure may not be conducted at the unit level 1 and level 2, whereas such procedure are often performed at unit level 3. ^22^ However, we did not find any difference in knowledge between the three unit levels. Although most of the treatment capacity in the level 1 unit is for normal babies, knowledge of signs of severity and early treatment is important at all levels. ^36^

Additional training with skill demonstrations and scenarios using mannequins have been proven to increase the level of knowledge of nurses, doctors, resident doctors, and specialists in Northern Nigeria. ^19^ To increase personal experience, healthcare practitioners need to practice each step of resuscitation. ^37^ Routine training may be an important indicator in determining the hospital's readiness to conduct the neonatal resuscitation. ^38^ Training including various steps of neonatal resuscitation, especially palpating umbilical cord pulse, endotracheal suctioning, endotracheal intubation, umbilical catheter placement, taking blood through an umbilical vein catheter, administering drugs/fluids through an umbilical catheter, and interpreting neonatal blood gas results, must be a priority and requires more intense training since most of the research subjects in this study rarely performed them. ^39^^,^^40^

Endotracheal intubation in neonates is rarely done because of the high level of difficulty and high risk of an adverse event for the procedure. ^40^^,^^41^ There is need of more time practicing and training to do endotracheal intubation. ^38^^,^^41^ The placement of an umbilical catheter, blood collection, and administration of drugs through the umbilical vein are rarely done, possibly because of the potential to be risk factors of sepsis. ^42^^,^^43^ More practice with evaluation are needed to increase the healthcare personnel confidence in doing the neonatal resuscitation. ^44^^–^^46^

### Research strengths and limitations

These findings may provide additional information to the guidelines of healthcare personnel training and qualifications. Nevertheless, several limitations exist in our study. First, the number of research subjects was reduced by the COVID-19 pandemic. We did consecutive sampling rather than random sampling which is more applicable for short time study. This low response rate may have biased the results. However, the participants joined this research voluntarily and were provided brief explanations to ensure comprehension of the questionnaire as an effort to decrease the bias. Second, we did not assess how many times the participants have joined the neonatal resuscitation training. Previous training is likely to be associated with the knowledge and experience score of the participants. Third, there is interaction between variables that may confound each other (i.e. profession with education, work experience, employee status, and care level). However, because of this collinearity, we cannot perform the multivariate analysis to distinguish the effect of each variable.

## Conclusion

The success of neonatal resuscitation is influenced by the readiness of the hospital, which can be assessed through indicators such as level of knowledge and experience of the healthcare personnel working at the hospital. In this study, we found that healthcare personnel from type A and type B hospitals were more experienced in conducting neonatal resuscitation than those from type D hospitals. We suggest that a type D hospital or other primary care should refer the neonate to a type A or type B hospital if there is sufficient time in cases of risk at need for resuscitation. Finally, larger observational studies with multicenter approaches should be conducted to substantiate our findings.

### Ethical considerations

This study was approved by the Ethics Committee of RSUD Dr. Soetomo Surabaya (Letter of Exemption 0335/LOE/301.4.2/II/2021).

## Data availability

### Underlying data

Figshare: Neonatal Resuscitation: Measuring The Readiness of Healthcare Personnel, https://doi.org//10.6084/m9.figshare.18865418. ^47^

The project contains the following underlying data:

• Experience.sav and
• Knowledge.sav.

Data are available under the terms of the Creative Commons Attribution 4.0 International license (CC-BY 4.0).
