# Supplementary material for: Neonatal resuscitation: A cross-sectional study measuring the readiness of healthcare personnel
Source: F1000Res. 2023 May 22;11:520. Originally published 2022 May 13. [Version 2] doi: 10.12688/f1000research.109110.2 (PMC10354456; doi:10.12688/f1000research.109110.2)
Supplement: Peer review history

# Peer review: Reviewer response for version 2

**DOI:** 10.5256/f1000research.147939.r174525

**Published:** 2023-07-18

**Authors:** Yellanthoor Ramesh Bhat

**Affiliations:** 1 Department of Paediatrics, Kasturba Medical College, Manipal Academy of Higher Education, Manipal, Karnataka, India

Authors revised the article and the revised version is accepted

Is the work clearly and accurately presented and does it cite the current literature?

Yes

If applicable, is the statistical analysis and its interpretation appropriate?

Not applicable

Are all the source data underlying the results available to ensure full reproducibility?

Yes

Is the study design appropriate and is the work technically sound?

Yes

Are the conclusions drawn adequately supported by the results?

Yes

Are sufficient details of methods and analysis provided to allow replication by others?

Yes

Reviewer Expertise:

Pediatrics, Neonatology, Intensive care, Ventilation

I confirm that I have read this submission and believe that I have an appropriate level of expertise to confirm that it is of an acceptable scientific standard.

# Peer review: Reviewer response for version 2

**DOI:** 10.5256/f1000research.147939.r174526

**Published:** 2023-06-05

**Authors:** Elisabeth M W Kooi

**Affiliations:** 1 University Medical Center Groningen, Beatrix Children's Hospital, Division of Neonatology, University of Groningen, Groningen, The Netherlands

I am satisfied with the responses by the authors and will endorse indexing of the paper.

Is the work clearly and accurately presented and does it cite the current literature?

Yes

If applicable, is the statistical analysis and its interpretation appropriate?

Partly

Are all the source data underlying the results available to ensure full reproducibility?

No

Is the study design appropriate and is the work technically sound?

Partly

Are the conclusions drawn adequately supported by the results?

Partly

Are sufficient details of methods and analysis provided to allow replication by others?

Yes

Reviewer Expertise:

NA

I confirm that I have read this submission and believe that I have an appropriate level of expertise to confirm that it is of an acceptable scientific standard.

# Peer review: Reviewer response for version 1

**DOI:** 10.5256/f1000research.120572.r140368

**Published:** 2022-07-13

**Authors:** Yellanthoor Ramesh Bhat

**Affiliations:** 1 Department of Paediatrics, Kasturba Medical College, Manipal Academy of Higher Education, Manipal, Karnataka, India

The article ‘Neonatal resuscitation: A cross-sectional study measuring the readiness of healthcare personnel” addresses the readiness of healthcare personnel with regard to the much needed topic on neonatal resuscitation. This study used questionnaire in 4 different types of hospitals. They found significantly higher experience scores among the healthcare personnel of type A and type B hospitals than the type D hospital. This need will help the local area to plan further training of personnel in type D hospital.

1. Overall the study appears reasonable. The information may help plan the policy regarding neonatal resuscitation and the target groups.
2. The first sentence, “ *Neonatal mortality is one of the standards of neonatal care*”-this sentence should be modified as ‘To decrease the neonatal mortality in developing countries, there is an urgent need to improve the neonatal care”.
3. Copyediting will be required throughout the manuscript.
4. Minimize the repetitions especially in methods and discussion.

Is the work clearly and accurately presented and does it cite the current literature?

Yes

If applicable, is the statistical analysis and its interpretation appropriate?

Not applicable

Are all the source data underlying the results available to ensure full reproducibility?

Yes

Is the study design appropriate and is the work technically sound?

Yes

Are the conclusions drawn adequately supported by the results?

Yes

Are sufficient details of methods and analysis provided to allow replication by others?

Yes

Reviewer Expertise:

Pediatrics, Neonatology, Intensive care, Ventilation

I confirm that I have read this submission and believe that I have an appropriate level of expertise to confirm that it is of an acceptable scientific standard, however I have significant reservations, as outlined above.

## References

[1] : Impact of education and training on neonatal resuscitation practices in 14 teaching hospitals in India. *Ann Trop Paediatr* .2001;21(1) :29-33 11284243

# Response

**Published:** 2023-05-07

**Authors:** Mahendra Tri Arif Sampurna

**Affiliations:** Faculty of Medicine Universitas Airlangga, Indonesia

We are very thankful for your availability and willingness to help us to become our reviewer on our manuscript entitled "Neonatal Resuscitation: A cross-sectional study measuring the readiness of healthcare personnel". We have revised our manuscript based point-on-point revision suggested.

**1. *"*** *The first sentence, “Neonatal mortality is one of the standards of neonatal care”-this sentence should be modified as ‘To decrease the neonatal mortality in developing countries, there is an urgent need to improve the neonatal care”."*

**Response:** Thank you for your valuable suggestion. We have revised the sentence to clear our research aim.

**2.** *"Copyediting will be required throughout the manuscript."*

**Response:** Thank you for your valuable suggestion. We have done copy editing using the help from professional English editing services.

**3.** *"Minimize the repetitions especially in methods and discussion."*

**Response:** Thank you for your valuable suggestion. We now have minimized the repetitions and keep the methods and discussion concisely.

# Peer review: Reviewer response for version 1

**DOI:** 10.5256/f1000research.120572.r137948

**Published:** 2022-07-11

**Authors:** Elisabeth M W Kooi

**Affiliations:** 1 University Medical Center Groningen, Beatrix Children's Hospital, Division of Neonatology, University of Groningen, Groningen, The Netherlands

This study is an important attempt to receive more insight in resuscitation readiness of various levels of staff in Indonesian hospitals of several levels. A questionnaire was developed and tested in ten nurses and then send out to a large number of hospital workers in neonatal departments. The results show a better readiness and experience in higher level hospitals, and some differences between characteristics of the participants.

 I have several concerns:

- The results are based on the responses of a relatively small part of the invited population. This low response rate may have biased the results which should be assessed and discussed in more detail. i.e.: Were certain levels of staff overrepresented?
- Also the validity of both questionnaires needs a critical reflection, including the assumed clinical relevant difference: even though there may be statistical significant differences, most of the time the difference between two groups is (only?) 1 or 2 points: is this relevant? The conclusion of the manuscript is fully based on this questionnaire that may need further validation including the assessment of a minimal clinical relevant difference.
- Also, are the questions based on an implemented national guideline that all neonatal caretakers should be aware of? How sure are the authors about the supposed correct answers to the questions, as some are consensus based?

Other concerns include:

- Have the authors assessed whether differences between subject characteristics were confounded by the other characteristics, i.e. were more men working in level A hospitals with more experience? This needs further analysis.
- Table 4 needs legends on what the various answers mean, apart from what is written in the main text.
- The abstract needs a brief explanation on the questionnaire (self-developed, ranges) in the methods section in order to be able to interpret the results section, Also, in my opinion it is not relevant to mention only p-values in the abstract results, but please include effect sizes and their uncertainties. Also response rate should be mentioned in abstract.
- I recognize a few language issues, i.e. what does " *divided the room into..."* mean? I would suggest to have the text reviewed on its details.
- In the conclusion the authors suggest to refer infants in need of resuscitation, I would rephrase into ‘risk at need for resuscitation’, otherwise referral will be too late. Also the authors conclude that more training is needed. Although this may very well be true, it is not what was investigated in this study, which is why I would weaken this statement.

Is the work clearly and accurately presented and does it cite the current literature?

Yes

If applicable, is the statistical analysis and its interpretation appropriate?

Partly

Are all the source data underlying the results available to ensure full reproducibility?

No

Is the study design appropriate and is the work technically sound?

Partly

Are the conclusions drawn adequately supported by the results?

Partly

Are sufficient details of methods and analysis provided to allow replication by others?

Yes

Reviewer Expertise:

Neonatology

I confirm that I have read this submission and believe that I have an appropriate level of expertise to confirm that it is of an acceptable scientific standard, however I have significant reservations, as outlined above.

# Response

**Published:** 2023-05-07

**Authors:** Mahendra Tri Arif Sampurna

**Affiliations:** Faculty of Medicine Universitas Airlangga, Indonesia

We are very thankful for your availability and willingness to help us to become our reviewer on our manuscript entitled "Neonatal Resuscitation: A cross-sectional study measuring the readiness of healthcare personnel". We have revised our manuscript based point-on-point revision suggested.

**1.** " *The results are based on the responses of a relatively small part of the invited population. This low response rate may have biased the results which should be assessed and discussed in more detail. i.e.: Were certain levels of staff overrepresented?"*

**Response:** Thank you for your valuable comment. We notice that our small sample may introduce the bias to our study. However, in this study we are trying to get the sample during a specific period to make sure there is no time bias. We have added mentions about this issue on the strength and limitation subsection.

**2.** *"The validity of both questionnaires needs a critical reflection, including the assumed clinical relevant difference: even though there may be statistical significant differences, most of the time the difference between two groups is (only?) 1 or 2 points: is this relevant? The conclusion of the manuscript is fully based on this questionnaire that may need further validation including the assessment of a minimal clinical relevant difference."*

**Response: **Thank you for your valuable suggestions. We use the validated questionnaire from prior publication of Jukkala et al in 2007. We confirm that the questions from that paper are suitable and relevant to our research aim. We also have added the discussion regarding the specific point of the questionnaire (in example : the lack of skill on endotracheal intubation) to enrich our discussion.

**3.** *"Are the questions based on an implemented national guideline that all neonatal caretakers should be aware of? How sure are the authors about the supposed correct answers to the questions, as some are consensus based?"*

**Response:** Thank you for your valuable comment. In our country, there is a need for neonatal resuscitation certificates to become a neonatal intensive care unit health provider. They will get the training to make sure of their competency in neonatal resuscitation. We confirm that questions which we obtained from Jukkala et al were suitable to assess the knowledge and skill of neonatal intensive care unit health providers.

**4.** *"Have the authors assessed whether differences between subject characteristics were confounded by the other characteristics, i.e. were more men working in level A hospitals with more experience? This needs further analysis."*

**Response: **Thank you for your valuable suggestion. We now have analyzed that there are variables that confounded others. The Profession may be related with Education, Work Experience, Employee Status, and Care Level. Age was also related to work experience. However, because of this collinearity, we cannot perform the multivariate analysis to distinguish the effect of each variable. We are trying our best to explain this issue in the results.

**5.** *"Table 4 needs legends on what the various answers mean, apart from what is written in the main text."*

**Response:** Thank you for your valuable suggestion, we now have added the information on Table 4.

**6.** *"The abstract needs a brief explanation on the questionnaire (self-developed, ranges) in the methods section in order to be able to interpret the results section. Also, in my opinion it is not relevant to mention only p-values in the abstract results, but please include effect sizes and their uncertainties. Also the response rate should be mentioned in the abstract."*

**Response:** Thank you for your valuable suggestion, we now have added a brief explanation about the questionnaire and included effect sizes in the abstract.

**7.** *"I recognize a few language issues, i.e. what does "divided the room into..." mean? I would suggest having the text reviewed on its details."*

**Response:** Thank you for your valuable suggestion. We have done copy editing using the help from professional English editing services.

**8.** *"In the conclusion the authors suggest referring to infants in need of resuscitation, I would rephrase into ‘risk at need for resuscitation’, otherwise referral will be too late. Also the authors conclude that more training is needed. Although this may very well be true, it is not what was investigated in this study, which is why I would weaken this statement."*

**Response:** Thank you for your valuable comment. We agree and have revised our conclusion. Again, thank you for your help.
